# Editorial: Current insights in sperm motility and male fertility

**DOI:** 10.3389/fendo.2026.2004991

**Published:** 2026-10-12

**Authors:** Gabriela R. Mendeluk, Nafisa H. Balasinor

**Affiliations:** 1 Laboratory of Male Fertility, Buenos Aires University Clinical Hospital “José de San Martín”, Instituto de Fisiopatología y Bioquímica Clínica (INFIBIOC), Department of Clinical Biochemistry, Faculty of Pharmacy and Biochemistry, University of Buenos Aires, Buenos Aires, Argentina; 2 National Institute for Research In Reproductive Health (ICMR-NIRRH), Bombay, India

**Keywords:** male infertility, astenozoospermia, basic research, biophysics, translational medicine

Asthenozoospermia, defined as a significant reduction in the proportion of motile spermatozoa, remains the most frequent diagnostic finding in the management of male infertility. Progressive motility is a hallmark attribute of the human male gamete and hence is a critical determinant of natural conception and an important predictor of treatment success in low-complexity assisted reproductive technologies (ARTs), particularly intrauterine insemination (IUI). Despite its pivotal role in diagnosis and function, sperm motility is increasingly compromised by modern environmental exposures and lifestyle choices. Factors such as sedentary habits, an unbalanced diet high in processed fats, chronic stress, and cigarette smoking negatively impact spermatogenesis, leading to concomitant reductions in sperm count, structural integrity, and overall semen quality.

Reflecting rigorous editorial standards and peer review, a total of 27 original manuscripts were submitted to this Research Topic, of which 6 high-quality studies were ultimately accepted (representing an acceptance rate of 22%).

These six contributions collectively cover a broad spectrum of basic, translational, and clinical reproductive science. The collection includes two clinical studies with direct implications for sperm motility management. The first article investigates a novel molecular post-translational pathway, the human sperm lactylome, in varicocele-associated asthenozoospermia. The second study addresses the diagnostic and therapeutic utility of seminal plasma Osteopontin for sperm motility. Moving from clinical pathology to molecular pathophysiology, this Special Issue builds upon the established role of oxidative stress in male infertility and elucidates the role of the gut microbiota-testis axis in sperm motility. The collection also includes a review of the adverse gonadotoxic effects of imatinib therapy, a crucial area of inquiry given that chronic myeloid leukemia frequently presents in men of reproductive age who wish to preserve their fertility potential. The final contribution is a biophysical, functional study that translates laboratory kinetics directly into actionable protocols for low-complexity ART procedures.

Summary of the Included Contributions

Cao et al. characterized the human sperm lactylome, demonstrating a disrupted lactate–lactylation axisin varicocele-associated asthenozoospermia. By analyzing paired pre- and post-varicocelectomy samples, the authors identified that decreased intracellular lactate levels directly correlate with global midpiece and flagellar hypolactylation. Quantitative proteomic profiling revealed 133 differentially lactylated sites on proteins essential for sperm motility, mitochondrial energy production, and axonemal structural organization, many of which partially recovered following surgical repair. By uncovering this novel, metabolite-driven, post-translational modification, the study provides a vital mechanistic foundation for understanding varicocele-induced sperm dysfunction and highlights prospective therapeutic targets.

Zhang et al. assessed the diagnostic and functional significance of seminal plasma osteopontin (OPN) in infertile men presenting with asthenozoospermia. A significant decrease in OPN concentrations in the infertile cohort was observed, establishing seminal OPN as a robust biomarker. Furthermore, *in vitro* supplementation with recombinant OPN induced clear, dose-dependent improvements in progressive motility, total motility, and specific kinematic parameters (VCL, VSL, and VAP), suggesting its potential utility as a therapeutic additive in ART media during sperm preparation.

Hu et al. investigated the gut-testis axis, demonstrating that Morinda officinalis oligosaccharides (MOS) effectively rescue sperm motility and reduce morphological abnormalities in a mouse model of asthenozoospermia. Mechanistically, oral administration of MOS successfully remodels the gut microbiota by selectively increasing the abundance of short-chain fatty acid-producing bacteria, notably the genus Anaerococcus. Fecal microbiota transplantation confirmed that Anaerococcus enrichment directly drives testicular protection, highlighting the therapeutic potential of targeting the gut microbiome in male factor infertility.

Wei et al. examined the multifaceted impact of oxidative stress in patients with asthenozoospermia undergoing *in vitro* fertilization (IVF). Sperm DFI, ROS levels, and MMP are indicators of sperm genetic stability, oxidative stress status, and energy metabolism level, respectively. Measuring these parameters provides significant insight into a deeper understanding of sperm function, including sperm motility. Crucially, the study showed that, while individual DFI or MMP evaluations yield only moderate predictive value for pregnancy, combining DFI and MMP assessments into a unified functional panel significantly enhances the predictive accuracy of fertilization rates, blastocyst development, and clinical pregnancy outcomes in ART settings, offering clinicians a robust diagnostic tool.

Ji et al. presented a comprehensive scoping review evaluating imatinib-induced testicular toxicity and male fertility impairment. The authors detailed how targeted tyrosine kinase inhibition (specifically blocking c-KIT and PDGFR signaling) triggers PI3K/AKT-mediated germ cell apoptosis, meiotic arrest, and structural flagellar defects downstream. Both experimental and clinical data identified the neonatal and prepubertal periods as particularly vulnerable developmental windows, during which exposure is frequently associated with partially irreversible impairment of spermatogenesis and endocrine function and hence fertility.

Mendeluk et al. established a novel, standardized diagnostic protocol utilizing 10-μm counting chambers and Computer-Assisted Sperm Analysis (CASA) to quantify Transition Hyperactivated (TH) sperm motility as a functional biomarker for male fertility. Evaluation of semen samples revealed that baseline TH levels (%THE) in ejaculates were significantly higher in fertile men compared to infertile patients. The authors identified a threshold of %THE > 9% as predictive of superior sperm recovery following density gradient or swim-up processing. Additionally, *in vitro* non-genomic L-thyroxine (T4) treatment significantly boosted TH motility and total gamete recovery in borderline samples, enabling previously sub-optimal ejaculates to successfully meet the threshold required for Intrauterine Insemination (IUI).

## Future directions

While the contributions compiled in this Research Topic significantly advance our current understanding of sperm physiology and clinical diagnostics, several core goals originally set forth in our call for articles remain unfulfilled, defining clear priorities for future research in the field:

International Consensus and Analytical Standardization: Achieving a globally accepted consensus on the standardized assessment of sperm motility and kinetic classification (velocity and linearity) remains an urgent need. Future initiatives must prioritize multicenter collaborative trials aimed at standardizing CASA hardware settings, chamber depths, frame acquisition rates, and mathematical algorithms across international laboratories to guarantee intercenter reproducibility.

Standardization of Capacitation and Hyperactivation Protocols: While progress has been made in baseline kinetic profiling, laboratory methodologies for standardizing hyperactivated motility under physiological capacitation conditions require rigorous refinement. Establishing standardized protocols for inducing, measuring, and interpreting hyperactivation *in vitro* will be essential for integrating functional sperm testing into routine clinical practice.

Translational Molecular and Biophysical Insights: A deeper exploration of the structural, mechanical, and bioenergetic signaling principles that govern flagellar motion is necessary. Bridging basic biophysical research—such as post-translational lactylation profiling and microfluidic motility selection—with clinical reproductive medicine will be vital to developing targeted pharmacological therapies for severe asthenozoospermia.

